# Methodological Biases in CBT Trial—Commentary: Modular Cognitive-Behavioral Therapy for Affective Symptoms in Young Individuals at Ultra-High Risk of First Episode of Psychosis: Randomized Controlled Trial

**DOI:** 10.3389/fpsyt.2020.00394

**Published:** 2020-07-28

**Authors:** Paolo Fusar-Poli, Joaquim Radua, Peter J. McKenna, Keith Laws, Cathy Davies, Sameer Jauhar

**Affiliations:** ^1^ Early Psychosis: Interventions and Clinical-detection (EPIC) Lab, Department of Psychosis Studies, Institute of Psychiatry, Psychology & Neuroscience, King’s College London, London, United Kingdom; ^2^ OASIS Service, South London and Maudsley NHS Foundation Trust, London, United Kingdom; ^3^ Department of Brain and Behavioral Sciences, University of Pavia, Pavia, Italy; ^4^ National Institute for Health Research, Maudsley Biomedical Research Centre, South London and Maudsley NHS Foundation Trust, London, United Kingdom; ^5^ Institut d’Investigacions Biomèdiques August Pi i Sunyer (IDIBAPS), Mental Health Research Networking Center (CIBERSAM), Barcelona, Spain; ^6^ Centre for Psychiatric Research and Education, Department of Clinical Neuroscience, Karolinska Institutet, Stockholm, Sweden; ^7^ Investigación Germanes Hospitalàries Research Foundation, Barcelona, Spain; ^8^ Department of Psychology and Sports Sciences, School of Life and Medical Sciences, University of Hertfordshire, Hatfield, United Kingdom; ^9^ Department of Psychological Medicine, Institute of Psychiatry, Psychology and Neuroscience, King’s College London, London, United Kingdom

**Keywords:** prevention, psychosis, clinical high risk (CHR), cognitive behavioural therapy (CBT), bias

Preventive interventions in individuals at clinical high risk for psychosis (CHR-P) are the mainstream approach to improve outcomes of the most severe mental disorder (1). Recent independent network (2, 3) or pairwise meta-analyses (4) converged indicating that there is no evidence to favour the recommended first-line treatment—Cognitive Behavioural Therapy, CBT—over other interventions (including needs-based-interventions) for the prevention of psychosis in CHR-P populations. Since the meta-analytical confidence interval of these estimates is large, there is high uncertainty (5) and future randomised controlled trials (RCT) are thus expected to have a major impact on the level of the evidence.

The article by Pozza and Dèttore (6) published in the past month, reports a new RCT of CBT for CHR-P individuals, which concluded in the abstract that CBT “can prevent psychosis risk and produce better outcomes on depression/anxiety than supportive intervention” (6). We highlight below a series of severe methodological problems which call into question the validity of some of these claims.

The authors indicate the RCT adhered to the CONSORT statement (7). However, CONSORT asks authors to state (point 23) the registration number and name of trial registry and to report the primary outcome in the abstract. Prospective registration, designed to increase transparency and decrease reporting bias, is also recommended by the International Committee of Medical Journal Editors (ICMJE) (8). It does not appear that this trial was registered, and no primary outcome is mentioned in the abstract.

Other departures from CONSORT guidelines include lack of specification of “who generated the random allocation sequence, who enrolled participants, and who assigned participants to interventions” (point 10). In this trial, randomisation was conducted by an independent researcher, who is not acknowledged, and ratings were carried out by two independent researchers on an internship, who are again not acknowledged. Similarly, while trial participants “were excluded by the consensus of a third independent assessor”, it is unclear who the assessor was.

Another issue is that the article lacks clarity over how at-risk designation was reached in the trial participants. To define CHR-P status, the authors employed the Comprehensive Assessment of At-Risk Mental States (CAARMS). In CHR-P trials, it is considered essential that the inter-rater reliability for the CAARMS is reported (9)—which is not the case here.

Additionally, to operationalize transition to psychosis from a CHR-P state, the authors employed DSM-5 and not CAARMS criteria. This is a departure from the standard approach in CHR-P trials. For example, in DSM, frank psychotic symptoms lasting one day and then remitting would qualify as full psychosis, under the category of DSM Brief Psychotic Disorders. Conversely, in the CAARMS, frank psychotic symptoms lasting up to seven days would still qualify for being at risk, under the CHR-P subgroup of Brief and Limited Intermittent Psychotic Symptoms (BLIPS) criteria (10). Relying on DSM rather than CAARMS criteria, the three patients meeting BLIPS criteria at baseline would already have been psychotic at this point, and so should have been excluded. Similarly, the five patients coded as “transitions” at outcome because they met DSM criteria for Brief Psychotic Disorder, if assessed with the CAARMS could have qualified as relapsed BLIPS—and therefore still be considered as at-risk/CHR-P (10).

The trial was small (n = 58) and underpowered. Recent meta-analyses published in this journal indicate that, assuming an efficacy risk ratio between experimental treatment and control conditions of 0.5, hundreds of CHR-P participants are needed to power an RCT testing preventive effects on transition to psychosis [Table 4 in Fusar-Poli et al. (11)]. Therefore, large multisite trials or risk enrichment strategies now seem to be the only way to properly investigate interventions. This is reflected in the Kaplan–Meier curve in **Figure 2** in Pozza et al. (6), which has a few large steps, indicating limited accuracy (12). Concerningly, no power analyses for any outcomes (including depression and anxiety) are reported.

In the Results, the authors reported the odds ratio between survival time of the CBT and control condition as being 0.37 (95% CI: 0.09–1.55), i.e. not significant. As noted above, it is unclear whether this was the primary outcome of the trial. Whether it was or not, failure to report such a negative finding in the abstract and failing to refer to it in the *Discussion* seems to amount to selective reporting (13). In fact, the (underpowered) Kaplan–Meier plot depicted in **Figure 2** of Pozza et al. (6) shows that treatment and control survival functions were close throughout most of the trial period, only diverging at the very far right (i.e. at 61 weeks). Kaplan–Meier survivor functions at the far right tend to be unreliable since fewer patients remain in the study group and the survival estimates are known to be less accurate (12). This type of error can be avoided if authors include the number of patients at risk (remaining subjects in the study) for each follow-up interval (12). Another way to control for this bias would be to plot the 95% confidence intervals for the Kaplan–Meier curves. Neither of these were done and instead the authors report a P = 0.05 (corrected down from P = 0.055) (14) far-right cumulative probability of developing psychosis as 12% (CBT) and 34% (control group).

In conclusion, this trial has weaknesses relating to measurement of outcomes (including the definition of transition), inadequate statistical power, incorrect interpretation of Kaplan–Meier outputs, selective reporting and failure to adhere to CONSORT guidance. This leads us to suggest that the main conclusion of this study, that CBT can prevent psychosis, needs to be amended. Some of these biases have been observed in previous CBT studies and have been amended [Retraction 1 (15) and Retraction 2 (16)].

## Author Contributions

PF-P and SJ conceived the study and drafted the first version of the manuscript; JR, PM, KL, and CD provided substantial contribution to the interpretation of the findings and revised the subsequent versions. All authors approve the current version of the manuscript.

## Conflict of Interest

SJ has received fees for educational talks given for Sunovian, and his employer (KCL) has received funding for educational talks he has given for Lundbeck. He is also a co-investigator on a drug trial for a compound manufactured by Alkermes.

The remaining authors declare that the research was conducted in the absence of any commercial or financial relationships that could be construed as a potential conflict of interest.
